# Ecological Model to Predict Potential Habitats of *Oncomelania hupensis*, the Intermediate Host of *Schistosoma japonicum* in the Mountainous Regions, China

**DOI:** 10.1371/journal.pntd.0004028

**Published:** 2015-08-25

**Authors:** Hong-Ru Zhu, Lu Liu, Xiao-Nong Zhou, Guo-Jing Yang

**Affiliations:** 1 Jiangsu Institute of Parasitic Diseases, Wuxi, People’s Republic of China; 2 Key Laboratory of Parasitic Disease Control and Prevention, Ministry of Health, Wuxi, People’s Republic of China; 3 Jiangsu Provincial Key Laboratory of Parasite Molecular Biology, Wuxi, People’s Republic of China; 4 National Institute of Parasitic Diseases, Chinese Center for Disease Control and Prevention, Shanghai, People’s Republic of China; 5 WHO Collaborating Center for Malaria, Schistosomiasis and Filariasis; Key Laboratory of Parasite and Vector Biology, Ministry of Health, Shanghai, People’s Republic of China; 6 Department of Epidemiology and Public Health, Swiss Tropical and Public Health Institute, Basel, Switzerland; 7 University of Basel, Basel, Switzerland; Australian National University, AUSTRALIA

## Abstract

**Background:**

Schistosomiasis japonica is a parasitic disease that remains endemic in seven provinces in the People’s Republic of China (P.R. China). One of the most important measures in the process of schistosomiasis elimination in P.R. China is control of *Oncomelania hupensis*, the unique intermediate host snail of *Schistosoma japonicum*. Compared with plains/swamp and lake regions, the hilly/mountainous regions of schistosomiasis endemic areas are more complicated, which makes the snail survey difficult to conduct precisely and efficiently. There is a pressing call to identify the snail habitats of mountainous regions in an efficient and cost-effective manner.

**Methods:**

Twelve out of 56 administrative villages distributed with *O*. *hupensis* in Eryuan, Yunnan Province, were randomly selected to set up the ecological model. Thirty out of the rest of 78 villages (villages selected for building model were excluded from the villages for validation) in Eryuan and 30 out of 89 villages in Midu, Yunnan Province were selected via a chessboard method for model validation, respectively. Nine-year-average Normalized Difference Vegetation Index (NDVI) and Land Surface Temperature (LST) as well as Digital Elevation Model (DEM) covering Eryuan and Midu were extracted from MODIS and ASTER satellite images, respectively. Slope, elevation and the distance from every village to its nearest stream were derived from DEM. Suitable survival environment conditions for snails were defined by comparing historical snail presence data and remote sensing derived images. According to the suitable conditions for snails, environment factors, i.e. NDVI, LST, elevation, slope and the distance from every village to its nearest stream, were integrated into an ecological niche model to predict *O*. *hupensis* potential habitats in Eryuan and Midu. The evaluation of the model was assessed by comparing the model prediction and field investigation. Then, the consistency rate of model validation was calculated in Eryuan and Midu Counties, respectively.

**Results:**

The final ecological niche model for potential *O*. *hupensis* habitats prediction comprised the following environmental factors, namely: NDVI (≥ 0.446), LST (≥ 22.70°C), elevation (≤ 2,300 m), slope (≤ 11°) and the distance to nearest stream (≤ 1,000 m). The potential *O*. *hupensis* habitats in Eryuan distributed in the Lancang River basin and *O*. *hupensis* in Midu shows a trend of clustering in the north and spotty distribution in the south. The consistency rates of the ecological niche model in Eryuan and Midu were 76.67% and 83.33%, respectively.

**Conclusions:**

The ecological niche model integrated with NDVI, LST, elevation, slope and distance from every village to its nearest stream adequately predicted the snail habitats in the mountainous regions.

## Author Summary

Schistosomiasis japonica is a parasitic disease caused by the infection of *Schistosoma japonicum*. *Oncomelania hupensis*, serving as the unique intermediate host of *S*. *japonicum*, has a distribution highly correlated with schistosomiasis epidemic. At present, elimination of *O*. *hupensis* is still an important target for disease control in the People’s Republic of China. In mountainous regions, compared with two other endemic regions, snails are hard to detect due to the complicated environmental conditions and poor transportation systems. In this study, we developed an ecological niche model to predict the potential habitats of *O*. *hupensis* using remote sensing data including vegetation index, land surface temperature, elevation, slope and the distance from every village to its nearest stream. Validation of the approach was performed in two counties with similar ecological conditions in Yunnan Province, P.R. China. Results revealed a model with a good consistency rate of 76.67% and 83.33% for the two counties, respectively. The model holds promise for snail surveillance in mountainous regions.

## Introduction

Schistosomiasis japonica, caused by *Schistosoma japonicum*, is transmitted by the snail intermediate host *Oncomelania hupensis* [1,2]. A reference group from the World Health Organization (WHO) reported that the disease burden associated with schistosomiasis was estimated to be 3.309 million disability-adjusted life years (DALYs) in 2010 [3,4,5,6]. In the People’s Republic of China (P.R. China), after more than 60 years’ effort, schistosomiasis was interrupted in five out of twelve endemic provinces and the infection rates of both human and livestock were reduced to no more than 5% countrywide [7,8]. However, the disease is still endemic in seven provinces in lake regions along the Yangtze River and in the mountainous region in western China [9]. In 2010, the Chinese government launched a schistosomiasis elimination programme with the goal of eliminating schistosomiasis as public health problem at the national level by 2015 [10].

It is widely acknowledged that the frequency and transmission dynamics of *S*. *japonicum* is closely related to its unique intermediate host, *O*. *hupensis* [11,12,13,14]. To eliminate schistosomiasis, controlling snail populations through mollusciciding was considered as one of the integrated control measures in P.R. China [15,16]. However, due to the complicated environmental conditions, snails in mountainous regions are difficult to control completely [17,18]. For example, recurrence rates were 6.15% (16/260) in the counties where transmission of schistosomiasis had been interrupted and 32.81% (21/64) in the counties under control from 1999 to 2003, at the country level of P.R. China [19]. In the mountainous regions of western China, 33.33% (7/21) of counties with transmission control and 4% (1/25) of counties with transmission interruption in Sichuan Province were confirmed to have local disease transmission again in 2004 [20]. In Chuxiong Autonomous Prefecture of Yunnan Province, the cumulative recurrence of snail infested areas were 1.882 km^2^ from 1994 to 2011 [21]. What’s more, the infection cases of snails are easily missed, so the endemic situation is inevitably underestimated in surveys of mountainous regions [22].

The question of “where snail infestations may be found in a certain mountainous region” is an urgent need in the schistosomiasis elimination stage in order to improve the surveillance and response system locally [23]. To address this question, it would be useful to understand the snail ecology in the mountainous region, leading to the development of a method to detect the habitats of *O*. *hupensis* promptly and precisely in a cost-effective manner. Previous studies found that the distribution of *O*. *hupensis* was strongly influenced by geographical and environmental characteristics [24,25]. Normalized difference vegetation index (NDVI) and land surface temperature (LST) were considered to be most successful environmental factors for snail habitat prediction [14,26]. Another important ecological feature of *O*. *hupensis*, as an amphibious snail, is the focal distribution along the water network, such as rivers, streams, etc. [27,28,29]. This is of concern because surface water serves as the most important indicator of the habitat of the amphibious snail [5]. In particular, water-flow in mountainous region is determined based on the elevation of the environmental settings. Therefore, the digital elevation model (DEM) was used to simulate the surface stream network and calculate slope data rapidly and precisely to enable use of important ecological metrics in the prediction of the snail habitats.

The ecological niche model is frequently used to predict the geographic distribution of a species [30,31,32]. For a certain species, it connects the distribution information and related environmental factors to reveal the relationship between them, and then predicts the distribution or potential habitats of the species. It has been widely applied in the research of animal habitat’s predictions, which were proved simple and convenient [14,33,34,35,36]. In this study, we aimed to identify *O*. *hupensis* habitats in the mountainous regions by ecological niche modeling based on various remote sensing derived data, i.e. NDVI, LST, elevation, slope and distance from every village to its nearest stream, so as to contribute to the development of surveillance tools for the national elimination programme of schistosomiasis and other snail-borne infectious diseases [37].

## Materials and Methods

### Study sites

Eryuan County is located in Yunnan Province, southwest of P.R. China, extending 25.80°-26.43° N and 99.54°-100.34° E. *S*. *japonicum* has been endemic there for more than 80 years based on historical records [38,39]. Up until 2012, the historically snail-infested areas covered 43.32 km^2^ [40]. According to the records of snail survey before year 2011, 56 of 90 administrative villages are historically endemic areas of *S*. *japonicum*. In this study, 12 villages with snail presence data were randomly sampled from 56 endemic villages to develop an ecological niche model.

The model validation was carried out in 30 villages out of the rest of 78 villages (12 villages selected for building model were excluded from the villages for validation) in Eryuan and 30 out of 89 villages in Midu (100.32°-100.78° E, 24.78°-25.53° N). Midu County is located about 120 km southeast of Eryuan and has similar ecological conditions as Eryuan. Among a total of 89 administrative villages in Midu County, 41 were endemic with schistosomiasis historically. The transmission of schistosomiasis in Midu County was interrupted in 1994, but the infested areas of *O*. *hupensis* rapidly relapsed after 1997. Up until 2008, the historically accumulative snail-infested areas covered 24.01 km^2^ in Midu County [41].

All study villages were selected by a chessboard method [42].

### Data collection and preparation

Remote sensing images covering Eryuan and Midu were downloaded from the National Aeronautics and Space Administration (Available at: http://reverb.echo.nasa.gov/). NDVI and LST were retrieved from the Moderate Resolution Imaging Spectroradiometer (MODIS), ranging from July 2002 to July 2011 with a temporal interval of eight days and sixteen days, respectively, and a spatial resolution of one kilometer. The Digital Elevation Model (DEM) was retrieved from the Advanced Space borne Thermal Emission and Reflection Radiometer (ASTER) with a spatial resolution of 30 meters. Slope, elevation and the distance from each village to its nearest stream were derived from DEM by the hydrological feature-based model in ArcGIS 10.0 (ESRI, Redlands, CA, USA).

Coordinates and snail presence data of 12 villages for model building in Eryuan County as well as digital administrative boundary data of Eryuan and Midu Counties at a scale of 1: 50,000 were collected from the local Schistosomiasis Control Station. All digitized data related to field data were imported into ArcGIS to construct a GIS database.

### Ecological niche model development

**Fig 1:**
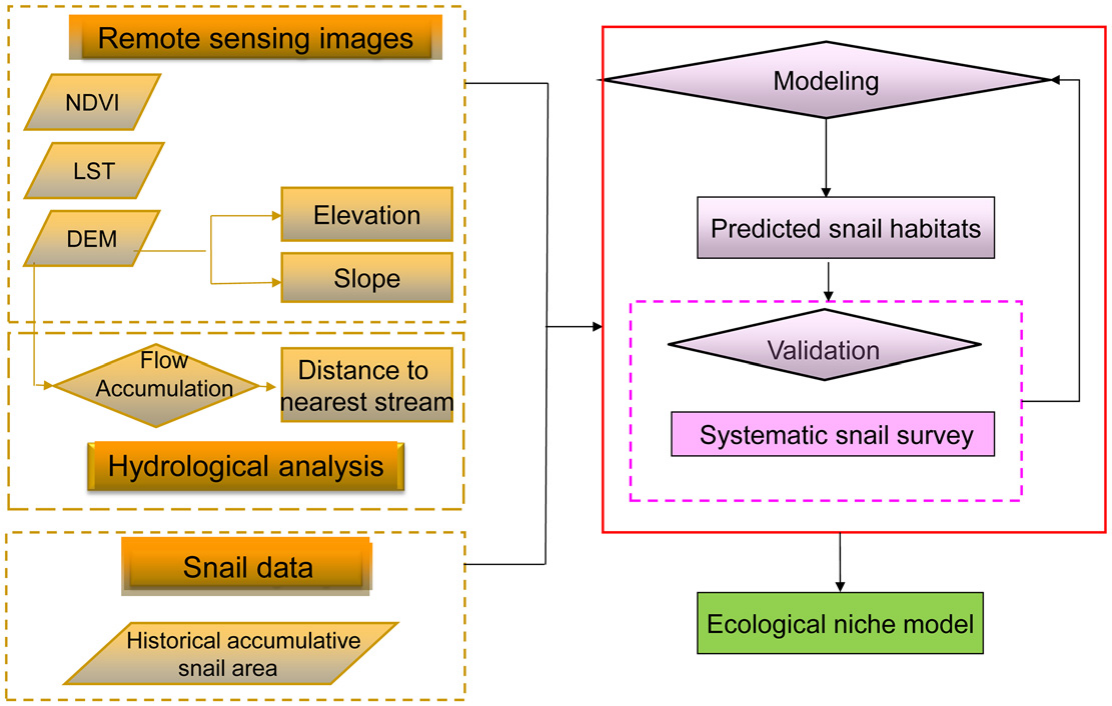
The flow chart of the study.

All remote sensing images (NDVI, LST and DEM) were pre-processed in ENVI 4.7 (The Environment for Visualizing Images), i.e. setting projection, mosaicking and extracting region of interest. The data of 9-year-averaged NDVI and LST for each pixel or cell in remote sensing images of Eryuan and Midu were calculated by the “Band Math” function. Then the remote sensing images of LST and NDVI over nine years were compiled into one image with annual data. The hydrological feature-based model was developed mainly by “Hydrological analysis” in ArcGIS 10.0 with the process of flow direction, flow accumulation, and stream net (Fig 1). The stream net could be generated by the Raster Calculator tool, e.g. “Flow accumulation ≥ 2,500”. Cells that have a flow accumulation beyond the threshold value were identified as stream net.

In 12 villages for model development in Eryuan County, the range (difference between maximum and minimum values) of environmental factors were extracted, namely NDVI, LST, slope, elevation and the distance from every village to its nearest stream and the *O*. *hupensis* suitable survival conditions of environment factors for habitats were defined according to ecological knowledge of snails.

Based on the suitable ranges of environmental factors for snail habitats, remote sensing images in Eryuan and Midu were extracted using the tools of “reclassification”. After that, a snail potential habitats map for each environmental factor covering Eryuan and Midu Counties was produced. Finally, all these five prediction maps were overlaid together in ArcGIS 10.0, and the prediction potential habitats was extracted by “Map Algebra”-“Raster calculator” [con((NDVI ≥ 0.446) & (LST ≥ 2.70) & (elevation ≤ 2,300) & (slope ≤ 11) & (distance ≤ 1,000), 1)], which meets the requirements for snail survival under five environmental conditions simultaneously.

### Validation of the ecological niche model

Model validation was carried out by snail survey at 30 administrative villages each in Eryuan and Midu, respectively, which were randomly selected via the chessboard method [43]. In each validation village, 30 sites were chosen along ditches and farmland [44] and snail surveys were performed at 10 m intervals in each snail collecting site, where a square frame measuring 0.11 m^2^ (33.3 cm × 33.3 cm) was placed. All snails within the frame in 30 collecting sites were collected into envelopes, labeled with location ID, and then recorded the presence situations of snails in each validation village into a table. Comparing the prediction potential habitats map and the field investigation results, the sensitivity, specificity and the consistency rate of the ecological niche model were calculated. The consistency rate was calculated according to eq (1):

$$\text{Consistency rate }=\;\frac{A_{n}+D_{n}}{A_{n}+B_{n}+C_{n}+D_{n}}\times 100\%$$ (1)

Where A denotes the number of validation villages with snail presence in the predicted suitable area, B denotes the number of validation villages with snail presence in the predicted unsuitable area, C denotes the number of validation villages without snail presence in the predicted suitable area, and D denotes the number of validation villages without snail presence in the predicted unsuitable area. Besides, “n” denotes the code of counties (n = 1, Eryuan County; n = 2, Midu County).

The National Institute of Parasitic Diseases, China CDC (IPD), Eryuan and Midu schistosomiasis control stations facilitated and validated field work methods and results.

## Results

### Data preprocessing

The GIS database of the study area was established including remote sensing images, historical snail data, digital administrative boundary files, digitized locations of endemic villages. A total of 108 monthly images and a 9-year-averaged image of NDVI and LST were generated, respectively.

### Ecological niche model development

**Fig 2:**
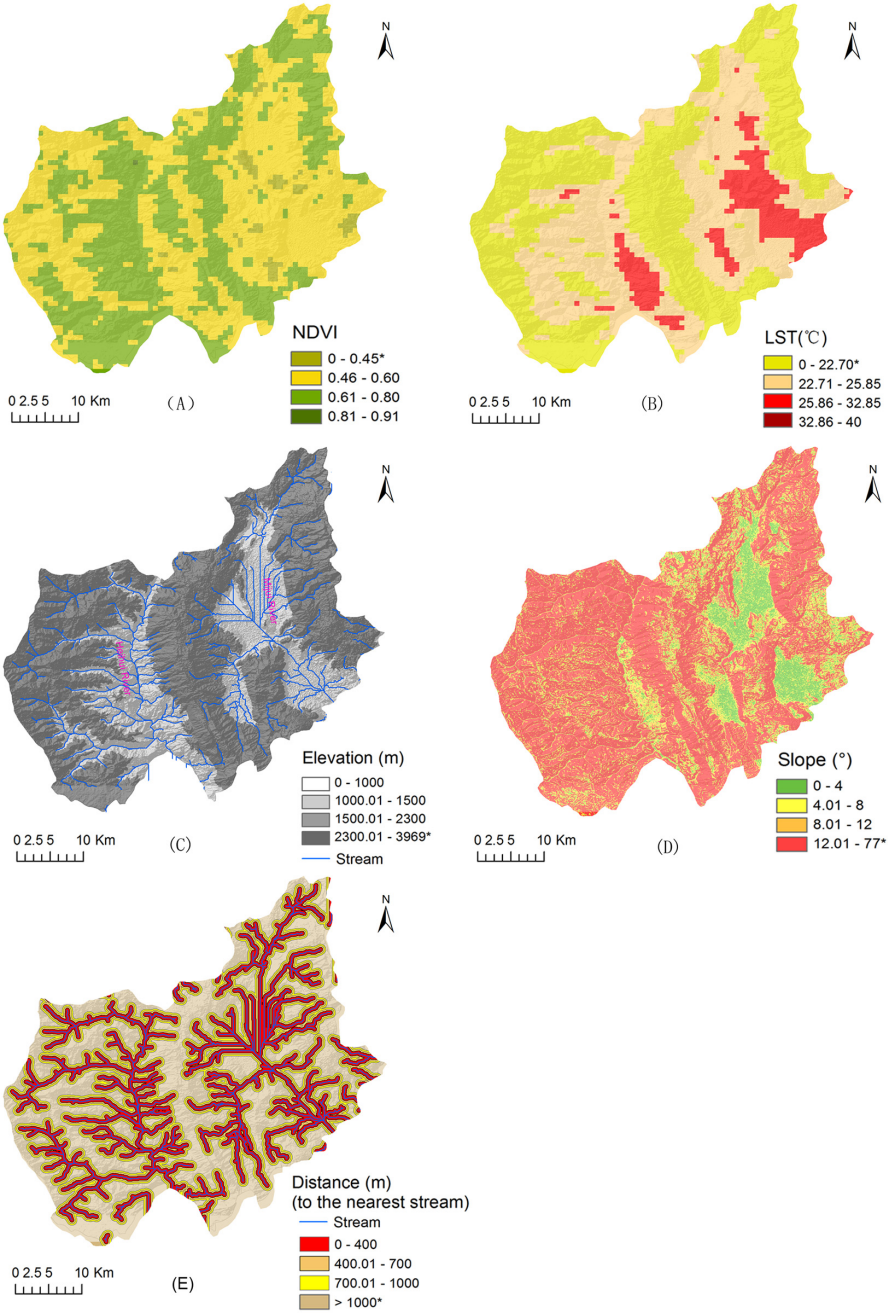
Processed remote sensing images of Eryuan County, Yunnan Province, China (* represents no *O*. *hupensis* in history).

As shown in Table 1, the ranges of environmental factors (difference between maximum and minimum values) of the 12 villages were extracted and the snail suitable conditions for habitats were defined according to biological characteristics of snails [1]: (i) NDVI in *O*. *hupensis* endemic areas was higher than 0.446, which was defined as the survival limit for *O*. *hupensis* (see Fig 2A). (ii) The lowest limit of LST was defined as 22.7°C. Therefore, two potential *O*. *hupensis* habitats were detected distributing in the east and west drainage basins (see Fig 2B). (iii) The water flow corresponded to two main streams: Miju River and Heihui River, both of which belong to the Lancang River basin. Elevation lower than 2,300 m was considered suitable for *O*. *hupensis* breeding (see Fig 2C). (iv) Slope less than 11° was treated as survival limit of *O*. *hupensis*, which mainly distributed in the east part of Eryuan (see Fig 2D). (v) Villages infested with *O*. *hupensis* were located less than 1,000 m away from the stream net (see Fig 2E).

**Table 1:** Environmental conditions for snail habitats in Eryuan County.

Factor	Mean	Range^^^	Suitable range^#^
**NDVI**	0.538	0.446–0.593	≥0.446
**LST** (°C)	25.53	22.70–27.72	≥22.70
**Elevation** (m)	2,085.2	1,898.0–2,227.0	≤2,300
**Slope** (degree)	4.55	1–11	≤11
**Distance** * (m)	324.6	28.0–915.1	≤1,000

*Distance: From every village to its nearest stream.

^^^Range: The difference between maximum and minimum values of environmental factors.

^#^Suitable range: The range of environmental factors which allow *O*. *hupensis* survive.

**Fig 3:**
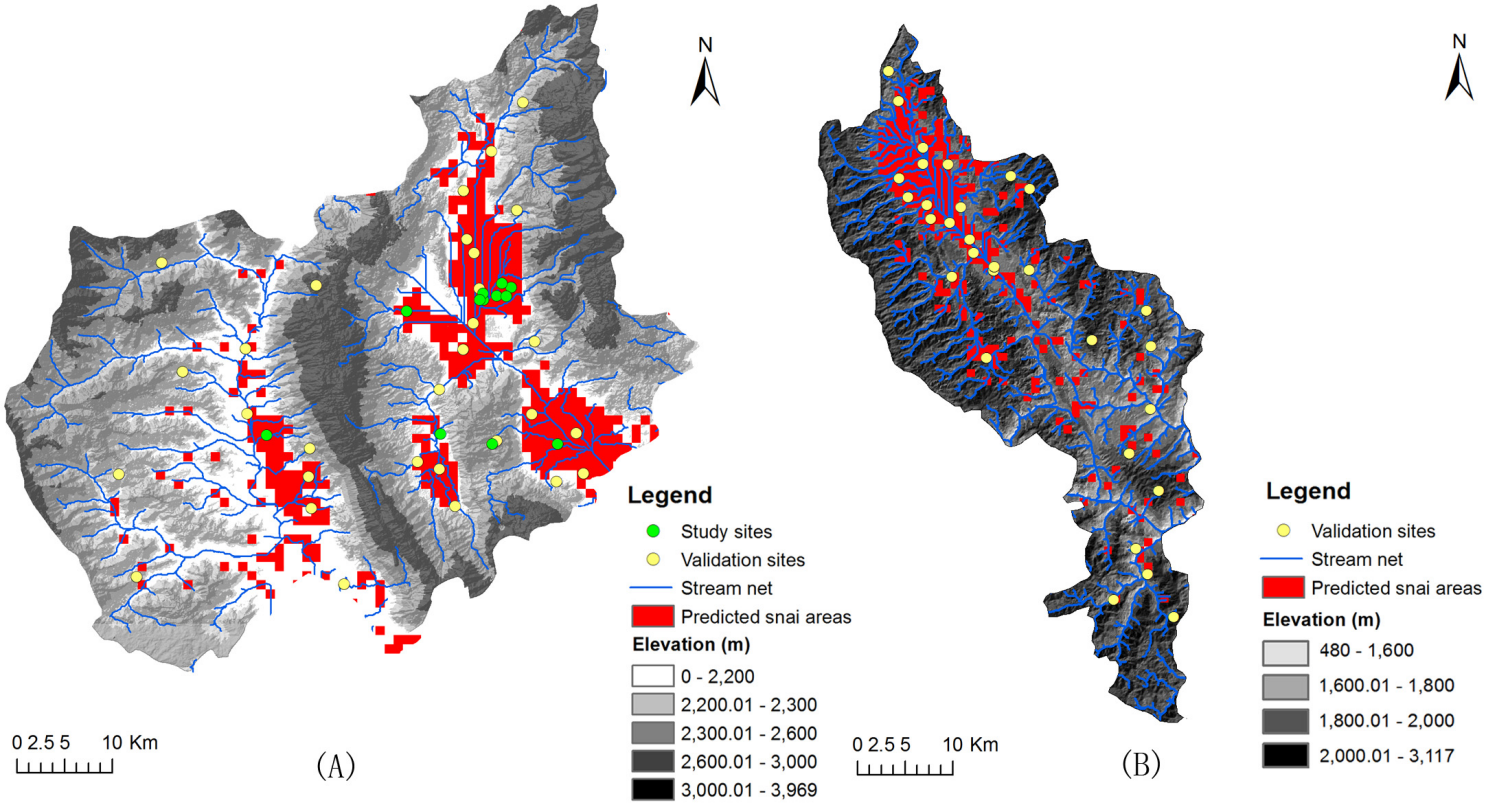
Prediction of snail infested areas in Eryuan (A) and Midu Counties (B), Yunnan Province, China.

Using the five aforementioned environmental indices as the parameters in the development of the ecological niche model simultaneously, the potential *O*. *hupensis* habitats in Eryuan and Midu were highlighted in red (Fig 3). We found that the potential *O*. *hupensis* habitats in Eryuan distributed in the Lancang River basin and *O*. *hupensis* in Midu showed a trend of clustering in the north and spotty distribution in the south.

### Model validation of efficiency

The validation for the ecological niche model was done in both Eryuan and Midu. As shown in Table 2, the situation of snail presence in each validation village was recorded and sorted into 4 categories (A_n_, B_n_, C_n_ and D_n_). In Eryuan and Midu, according to the field investigation, 23 and 25 validation villages (A_n_ + D_n_) had the same snail presence status with the predictions of the model, respectively. However, the results of field investigation in 7 and 5 validation villages were inconsistent with model prediction in Eryuan and Mindu, respectively (B_n_ + C_n_).

**Table 2:** Results of the field investigation for model validation in Eryuan and Midu Counties.

Model Prediction		Eryuan		Midu	
Validation		Suitable area	Unsuitable area	Suitable area	Unsuitable area
**Field Survey**	**Snail presence**	13 (A_1_)	3 (B_1_)	16 (A_2_)	3 (B_2_)
	**Snail absence**	4 (C_1_)	10 (D_1_)	2 (C_2_)	9 (D_2_)
	**Total**	17	13	18	12

In Eryuan, the sensitivity and specificity of the prediction were 76.5% (13/17) and 76.9% (10/13) respectively. By comparison, the two rates in the field survey of Midu were 88.9% (16/18) and 75.0% (9/12), respectively. The model consistency rates were 76.67% and 83.33% in Eryuan and Midu, respectively.

## Discussion

This study, innovatively using NDVI, LST, together with DEM, derived hydrological features (i.e., slope, elevation and the distance from every village to its nearest stream) to develop an environment niche model. Together these indices defined the potential habitats of *O*. *hupensis* in the mountainous endemic areas of schistosomiasis in China. It is the first time to use this model with the remarkable result that the predicted snail habitats had a good consistency rate of 76.67% and 83.33% in Eryuan and Midu, respectively. The data collection and preparation is relatively straightforward, can be updated in a timely way and is free of charge, which provides an advantage in the public health applications, particularly in the surveillance of and response to water-associated diseases.

Previous studies have proved that the distribution of *O*. *hupensis* is related to a close relationship with environment factors such as elevation, bodies of water, vegetation and temperature [24,27,44,45]. A study carried out in lake and marshland regions had found that low elevation was more suitable for survival of the snail than high elevation, which was consistent with our finding that *O*. *hupensis* survives below a ceiling elevation of 2,300 m [46]. Similarly, Chen found that the distances from 90% of the *S*. *japonicum* endemic counties to their nearest rivers are less than 1014 meters, which agreed with the finding of the “1,000 m buffer zone” in the present study [47]. However, most of the previous studies weren’t concerned with the multiple environmental factors found in mountainous regions. In this study, we integrated five environmental factors by developing an ecological niche model to predict snail habitats in the mountainous regions and this proved to be a cost-effective approach.

Recent georeferenced topographical maps could be used to digitize rivers and other water bodies, but changes in rivers is variable over time related to landscape changes, which prevents recognition of the potential watershed. Further, the climate in the mountainous regions of Yunnan Province is characterized by distinct rainy and dry seasons. The stream sectors with less flow rate may have no water flow during the dry season, but have flowing water in the rainy season. Such stream sectors are always neglected by the published hydrological maps, but they have significant influence on the distribution of *O*. *hupensis*. Recently, other researchers used hydrological models to predict mosquito abundance within watersheds or potential resurgence of *Schistosoma haematobium* [29,48,49]. Hydrological feature-based modeling is considered to be a powerful tool for determining the potential habitats of snail.

In this study, ASTER DEM was not only applied to extract the elevation and the slope but also to calculate the stream nets, which could show the potential water system hub effectively. The real temporary and even the potential streams could be investigated simultaneously with the assistance of different threshold settings. The relationship between the slope of mountainous areas and *O*. *hupensis* has been very rarely reported. In this study, we found that slope played an important role in the distribution of *O*. *hupensis*. Our field survey found that rice field terraces distributed anywhere in mountainous areas of Eryuan and Midu, were where *O*. *hupensis* was always found. The dams of terraces have a high slope value and flow velocity, which does not hold the water, and the flow velocity could be too fast for *O*. *hupensis* to maintain their existence in that region. In addition, steep areas are often used to plant economic trees, which impede the breeding of *O*. *hupensis*. Due to all these factors, the possibility for *O*. *hupensis* breeding decreases as the slope increases. Compared with scenes from Landsat TM or SPOT, NDVI and LST from MODIS have a relatively low resolution, but they are available free and easily accessed. However, the model can be improved in several ways. First, remote sensing is developing rapidly, and use of data with high resolution would be better, especially in “hot spots” of prediction. Second, in consideration of the complicated conditions in mountainous regions, the contribution of different influencing factors to the model can be calculated. Third, one shortcoming of the DEM is that the stream order and the elevation are correlated. For example, low flow orders are more likely to occur in the high areas and *vice versa*. Further research could be done to explore the prediction of *O*. *hupensis*, such as *O*. *hupensis* habitats in key irrigation canals and ditches with remote sensing images of higher resolution. Finally, in addition to environmental factors, social factors could be also considered.

We concluded that the model presented here can be used to predict potential *O*. *hupensis* habitats with a good consistency rate in mountainous regions. The model could become important tools for the prediction of *O*. *hupensis* in mountainous areas, particularly in areas where snail survey is a difficult task. We encourage other groups to adopt and further develop our prediction approach in different geographical areas in relation to other neglected tropical diseases to facilitate spatial targeting of controlled interventions in a timely and cost-effective manner.
